# Hippocampal circuits underlie improvements in self‐reported anxiety following mindfulness training

**DOI:** 10.1002/brb3.1766

**Published:** 2020-07-23

**Authors:** Gunes Sevinc, Jonathan Greenberg, Britta K. Hölzel, Tim Gard, Thomas Calahan, Vincent Brunsch, Javeria A. Hashmi, Mark Vangel, Scott P. Orr, Mohammed R. Milad, Sara W. Lazar

**Affiliations:** ^1^ Department of Psychiatry Massachusetts General Hospital Harvard Medical School Boston MA USA; ^2^ Department of Neuroradiology Klinikum rechts der Isar Technical University of Munich Munich Germany; ^3^ Department of Anesthesia, Pain Management & Perioperative Medicine Dalhousie University Halifax NS Canada; ^4^ Psychiatry Department New York University Grossman School of Medicine New York NY USA

**Keywords:** extinction, extinction retrieval, fear memory, fMRI, hippocampus, mindfulness, subiculum

## Abstract

**Introduction:**

Mindfulness meditation has successfully been applied to cultivate skills in self‐regulation of emotion, as it employs the unbiased present moment awareness of experience. This heightened attention to and awareness of sensory experience has been postulated to create an optimal therapeutic exposure condition and thereby improve extinction learning. We recently demonstrated increased connectivity in hippocampal circuits during the contextual retrieval of extinction memory following mindfulness training.

**Methods:**

Here, we examine the role of structural changes in hippocampal subfields following mindfulness training in a randomized controlled longitudinal study using a two‐day fear‐conditioning and extinction protocol.

**Results:**

We demonstrate an association between mindfulness training‐related increases in subiculum and decreased hippocampal connectivity to lateral occipital regions during contextual retrieval of extinguished fear. Further, we demonstrate an association between decreased connectivity and decreases in self‐reported anxiety following mindfulness training.

**Conclusions:**

The results highlight the role of the subiculum in gating interactions with contextual stimuli during memory retrieval and, also, the mechanisms through which mindfulness training may foster resilience.

## Graphical abstract

Mindfulness meditation has successfully been applied to cultivate skills in self‐regulation of emotion, as it employs the unbiased present moment awareness of experience. This heightened attention to and awareness of sensory experience has been postulated to create an optimal therapeutic exposure condition and thereby improve extinction learning. Here, we examine the role of structural changes in hippocampal subfields and further demonstrate an association between mindfulness training‐related increases in subiculum and decreased hippocampal connectivity to lateral occipital regions during contextual retrieval of extinguished fear. Further, we demonstrate an association between decreased connectivity and decreases in self‐reported anxiety following mindfulness training. These results highlight the role of the subiculum in gating interactions with contextual stimuli during memory retrieval and, also, the mechanisms through which mindfulness training fosters resilience.

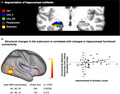

## 1 INTRODUCTION

The ability to learn and remember that a stimulus is no longer threatening is critical for healthy emotional functioning and lies at the heart of exposure therapies (Foa & McLean, 2016). During exposure, individuals are presented with fear‐inducing stimuli in a controlled environment until the response to the eliciting stimulus gradually declines while behavioral patterns of avoidance that reinforce the fear response dissolve (Craske & Mystkowski, 2006). Critically, mindfulness meditation creates conditions similar to, and optimal for, exposure by allowing aversive stimuli to be experienced with nonreactive acceptance (Hölzel et al., 2011; Treanor, 2011), and thereby facilitates extinction learning. In line with this overlap, we previously reported mindfulness training‐dependent increases in hippocampal functional connectivity during the retrieval of extinguished fear memories (Sevinc et al., 2019). Here, we further this work by examining the role of structural changes in hippocampal subfields on hippocampal connectivity during fear extinction retrieval.

Extinction learning consists of three phases: acquisition, consolidation, and retrieval (Quirk & Mueller, 2008). The hippocampus has been implicated in the consolidation and retrieval phases of extinction, especially in the utilization of contextual information and in gating the expression of either the conditioned fear or extinction memory during retrieval (Bouton, 1993; Hartley & Phelps, 2010; Milad, Quinn, et al., 2005; Milad, Wright, et al., 2007). While the role of hippocampus in context‐dependent retrieval of extinguished memory is now established (Milad, Wright, et al., 2007), the specific role of subfields is still being investigated (Rolls, 2016); and differential roles have been proposed both in humans (Dimsdale‐Zucker, Ritchey, Ekstrom, Yonelinas, & Ranganath, 2018) and in rodents (Ji & Maren, 2008; Lovett‐Barron et al., 2014). For instance, while both CA1 and CA3 have been implicated in the acquisition of context‐dependent extinction in rodents, only area CA1 has been found to be critical for contextual memory retrieval (Ji & Maren, 2008). Although differential roles have been proposed for each of the hippocampal subfields, the specific function of the subfield within in the context of mindfulness‐induced improvements in extinction learning is still unknown.

We have previously demonstrated hippocampal changes following mindfulness training [Greenberg et al., 2018; Holzel et al., 2011] and speculated that they may be due, in part, to increased synaptogenesis (Eriksson, 2003; Eriksson et al., 1998; Kim & Diamond, 2002). Others have reported differences in gray matter density within the hippocampus in long‐term meditation practitioners versus controls (Holzel et al., 2008; Luders, Toga, Lepore, & Gaser, 2009), and specifically within the subiculum (Luders, Kurth, Toga, Narr, & Gaser, 2013). With respect to mindfulness‐dependent changes in extinction retrieval, we have reported increased task‐dependent functional connectivity in areas associated with enhanced cognitive control and contextual processing during early phases of retrieval as a function of increases in hippocampal structure (Sevinc et al., 2019). While enhanced task‐dependent functional coupling of the hippocampus is in line with the retrieval associated improvements in contextual processing (Bar, 2007; Marstaller, Burianová, & Reutens, 2017; Spreng & Andrews‐Hanna, 2015), the functional role of the morphological changes in hippocampal subfields is yet to be investigated.

Here, we test the relationship between structural alterations in hippocampal subfields and hippocampal functional connectivity following mindfulness training using a 2‐day fear extinction and recall procedure. Based on previous findings of increased subiculum volume in long‐term meditation practitioners, compared to controls (Luders, Kurth, Toga, Narr, & Gaser, 2013), as well as its role in both memory retrieval (Ledergerber & Moser, 2017; Roy et al., 2017) and the regulation of stress response (Bannerman et al., 2004), we hypothesized that changes in subiculum following mindfulness training would be associated with changes in the functioning of hippocampal networks during retrieval. Given the relationship between hippocampal structure, anxiety, and emotion regulation (Bannerman et al., 2004; Herman, Dolgas, & Carlson, 1998), we additionally hypothesized an association between changes in hippocampal functioning and changes in self‐reported anxiety.

## 2 MATERIALS AND METHODS

Individuals between 18 and 50 years of age were recruited and were randomized to one of two stress‐reduction programs: Mindfulness‐Based Stress Reduction (MBSR) or Stress Management Education (SME) on a 2:1 ratio, stratified by gender. Participants were recruited via public transportation advertisements for stress‐reduction programs. The inclusion criteria were right‐handedness, no current psychiatric or neurological disorders, and not being engaged in psychotherapy or using psychotropic medications within 12 months prior to the study. Individuals with significant prior meditation experience were also excluded (having taken no more than 4 mind–body classes of any kind, for example, meditation, yoga, and tai chi, in the past 12 months or more than 10 classes in their lifetime), and individuals who endorsed any standard MRI safety exclusion criteria. The study was reviewed and approved by the Institutional Review Board of Partners HealthCare.

As reported in Sevinc et al. (2019), 94 participants completed initial testing and were randomized, 89 attended at least one class (58 MBSR, 31 SME), and 49 MBSR and 27 SME participants completed MRI scanning at the post‐time point. There were no differences between groups in terms of gender (MBSR: 28 female, 14 male; SME: 15 female, 10 male; *χ* ^2^ = 0.30, *p* = .58), age (MBSR 31.14 ± 7.71; SME 33.08 ± 18.02; *t* = −0.94, *p* = .35), or years of education (MBSR 17.40 ± 3.08, SME 18.02 ± 2.51; *t* = −0.84, *p* = .40). Total hours of home practice were 23.50 ± 10.87 hr for MBSR and 34.82 ± 19.74 hr for SME (*t* = 2.65, *p* = .013).

The 8‐week MBSR program (Kabat‐Zinn, 1990) consisted of weekly 2‐hr classes that included didactic teaching about mindfulness, experiential practice of mindfulness, and group discussions on impediments to effective practice as well as practical day‐to‐day applications of mindfulness. Formal meditation practices included body scan meditation, breath awareness meditation, and mindful yoga. The SME program, adapted from Hoge and colleagues (Hoge et al., 2013), consisted of 2‐hr weekly group sessions over 8 weeks. Didactic content included the effects of stress on health and optimizing one's personal health care, understanding positive coping behavior, optimizing nutrition to decrease stress, the role of exercise in reducing stress, sleep hygiene, and humor. Participants learned light strength training and aerobic exercises which they also practiced at home. Both classes included an additional 4‐hr class at the end of the 6th week. Both groups were instructed to practice at home for 40 min daily and were given audio recording to facilitate practice. The MBSR course was led by a certified MBSR instructor (Zayda Vallejo); the SME program was led by a licensed physical therapist (Jen Kelly).

Participants (MBSR *n* = 37; SME *n* = 22) completed the Perceived Stress Scale (PSS; Cohen, Kamarck, & Mermelstein, 1983), Spielberger State Trait Anxiety Inventory (STAI; Spielbeger, 1987), and the Mindful Attention Awareness Scale (MAAS) (Brown & Ryan, 2003). Differences between stress, mindfulness, and anxiety levels between groups were examined using analyses of covariance (ANCOVA) and independent samples *t* tests. The scanning protocol comprised a 2‐day classical fear‐conditioning and extinction procedure validated in several healthy (Milad & Quirk, 2002; Milad, Quirk, et al., 2007) and patient (Holt et al., 2009; Milad et al., 2009; Orr et al., 2006) populations. Briefly, the fear‐conditioning procedure consisted of acquisition (conditioning) and extinction phases on Day 1, and an extinction recall phase on Day 2. An electric stimulation, set to a "highly annoying but not painful" level for each participant, was applied to fingers on the left hand to create the unconditioned stimulus (US) during conditioning. Images of two different rooms provided contextual information. Each room contained a blue, red, or yellow lamp, which constituted the conditioned stimuli (CS). Selection of the CS+ and CS− colors was randomly determined and counterbalanced across participants and time points. During the conditioning phase, 2 CS (CS+) were paired with the 500‐msec US to the left hand at a partial reinforcement rate of 60%. One of these colors was subsequently extinguished during the extinction phase (CS + E), whereas the other CS+ was not (CS + U). The third color was never paired with a shock and served as the CS−. On Day 2, all three stimuli were presented without a shock. For each trial during the experiment, the virtual context was presented for 18 s: 6 s alone followed by 12 s in combination with a CS+ or CS−.

### 2.1 MRI data collection and analysis

Imaging data were acquired on a Siemen's Prisma 3.0 T equipped for echo planer imaging (EPI; Siemens Medical Systems) with a 32‐channel gradient head coil. An automated scout image was obtained to facilitate alignment of pre‐ and postintervention scans. High‐resolution three‐dimensional magnetization‐prepared rapid acquisition gradient echo (MPRAGE) sequences were acquired (repetition time [TR] = 2.53 ms, echo time [TE] = 1.74 ms, flip angle = 7°; 1 mm isotropic voxels, FOV = 256 cm, 176 axial slices). Functional images were acquired with gradient–echo T2*‐weighted sequences (TR = 3 s, TE = 30 ms, flip angle = 90°, FOV = 1,400*1,400; slice thickness = 2.5 isotropic voxels).

All participants were scanned within 2 weeks before and after the courses. MPRAGE data suitable for structural analysis included 38 MBSR and 23 SME participants. For reconstruction and segmentation of the brain, the longitudinal analysis stream in FreeSurfer software suite version 5.3 was used (Reuter & Fischl, 2011; Reuter, Rosas, & Fischl, 2010; Reuter, Schmansky, Rosas, & Fischl, 2012). No manual interventions were needed following a visual check of segmentation accuracy. Symmetrized percent changes in hippocampal volumes were extracted using FreeSurfer's “asegstats2table” and “long_stats_slopes” streams. Symmetrized percent change refers to the rate of change with respect to the average intensity between two time points rather than the rate of change from the first to the second time point and has been reported to be a more robust and sensitive measure (Reuter & Fischl, 2011).

Forty‐two MBSR participants and 25 SME participants had functional neuroimaging data available for functional connectivity analyses. Spatial preprocessing of functional volumes was done using SPM8 and included slice timing correction, realignment, normalization, and smoothing (6 mm FWHM Gaussian filter) (Welcome Department of Imaging Neuroscience; http://www.fil.ion.ucl.ac.uk/spm). Seed‐based functional connectivity analyses were performed using the weighted general linear modeling option in the CONN toolbox v.18b (Whitfield‐Gabrieli & Nieto‐Castanon, 2012). For time and frequency decomposition, a band‐pass filter [0.01–0.15] was applied. To address potential spurious correlations caused by head motion, artifact detection toolbox was used, as implemented in Conn toolbox. Additionally to address the confounding effects of participant movement, Conn toolbox utilizes the CompCor method (Behzadi, Restom, Liau, & Liu, 2007). T1‐weighted images are segmented, and principal components associated with segmented white matter and cerebrospinal fluid are used as temporal covariates and removed from the BOLD functional data using linear regression, along with realignment parameters. The resulting residual BOLD timeseries were band‐pass filtered using 0.008 Hz–0.09 Hz.

**Figure 1:**
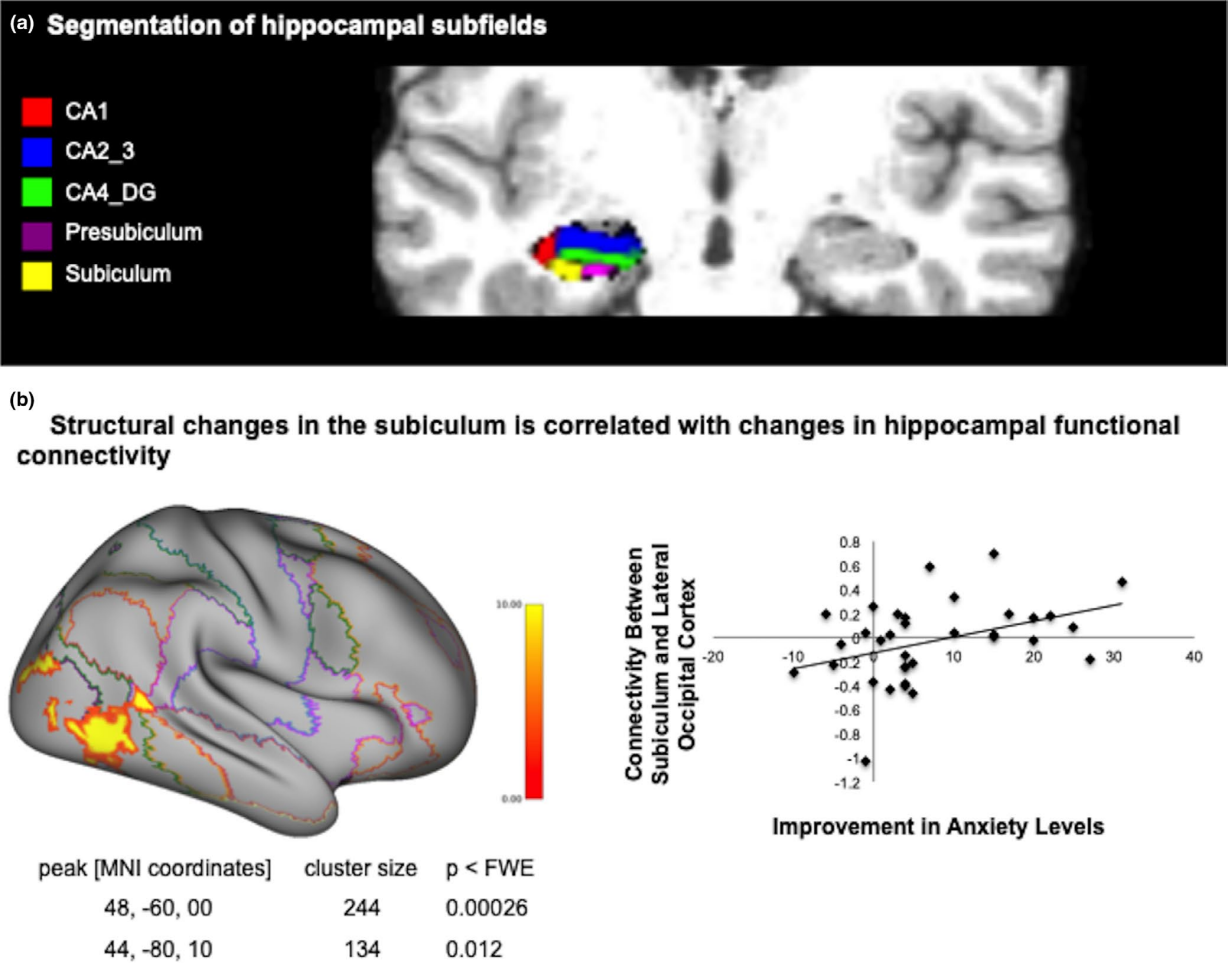
(a) Segmentation of hippocampal subfields was done using FreeSurfer 5.3 for the following subfields: presubiculum, CA1, CA2, fimbria, subiculum, CA4, and hippocampal fissure. The figure shows an axial view of an example T1‐weighted structural image and relevant segmentations. (b) The figure on the left shows the results of the seed‐based functional connectivity analysis, mapped onto Conte69 atlas via Connectome Workbench using trilinear interpolation. Volume increases within the subiculum from pre‐ to post‐MBSR are associated with decreased functional connectivity between the left hippocampus and two clusters in lateral occipital cortex (MNI coordinates [48, −60, 00], *k* = 244, and [44, −80, 10], *k* = 134). (b) shows the association between changes in connectivity and improvements in anxiety scores [post–pre] (*r* = .394, *p* = .021, *n* = 34) following mindfulness training. The X‐axis depicts the Fisher transformed correlation coefficient of connectivity between the left hippocampus and the reported cluster, while the Y‐axis depicts changes in anxiety levels [post–pre]

Relying on previous findings in the left hippocampus (Sevinc et al, 2019), the analyses focused on comparison of volume changes within the following subfields of the left hippocampus: presubiculum, CA1, CA2, fimbria, subiculum, CA4, and hippocampal fissure (see Figure 1a). For the functional connectivity analyses, an anatomically defined left hippocampal ROI was used. This ROI is part of the CONN toolbox default ROIs and is defined using the FSL Harvard–Oxford atlas. First‐level analyses were conducted using the Pearson's correlation coefficients between the residual blood oxygen level‐dependent (BOLD) time–course of the hippocampal seed and the time–course of all other voxels. The correlation coefficients were converted to normally distributed z‐scores using the Fisher transformation to improve the validity of second‐level general linear model analyses. The coefficients represent the relative degree of connectivity between each voxel and seed, and were subsequently used for second‐level analysis of relative functional connectivity as implemented in the CONN toolbox. These voxelwise statistics were conducted at *p* < .001 and were corrected using a cluster level using family‐wise error correction at *p* < .05.

## 3 RESULTS

### 3.1 Self‐reported outcomes measures

As previously reported (Sevinc et al., 2019), both interventions decreased levels of perceived stress (MBSR (*n* = 37) ΔPSS = 4.57 ± 8.04, *t*(36) = 3.45, *p* < .001, Cohen's *d* = 0.56, [CI: 1.89–7.25]; SME (*n* = 22) ΔPSS = 3.68 ± 6.52, *t*(21) = 2.65, *p* = .015, *d* = 0.57, [CI: 0.79–6.57]), with no statistical difference between groups in ΔPSS scores *t*(59) = −0.44, *p* = .66. There were no differences between the MBSR and SME groups in mindfulness (*F*(1,59) = 3.251, *p* = .77, partial *η* ^2^ = 0.55), and a marginally significant difference in anxiety levels [*F*(1,59) = 3.93, *p* = .052, partial *η* ^2^ = 0.63]. No correction was made for multiple comparisons.

### 3.2 Structural changes in hippocampal subfields

For MBSR, the symmetrized percent change values were as follows: Hippocampus(L), −0.746; presubiculum, −0.918; CA1, −1.144; CA2/3 −0.162; fimbria, −5.152; subiculum 0.073; CA4/DG 0.158; and hippocampal fissure, 1.474. For the SME, the symmetrized percent change values were as follows: the Hippocampus(L), −0.732; presubiculum, −1.068; CA1, −0.497; CA2/3 0.054; fimbria, −0.669; subiculum 0.073; CA4/DG 0.064; and hippocampal fissure, 1.722. Independent samples *t* tests revealed no significant differences between groups in terms of structural changes within the subfields.

### 3.3 Structural changes in hippocampal subfields and changes in hippocampal functional connectivity

As a second step, we examined associations between pre‐ and postintervention volume changes in hippocampal subfields and changes in functional connectivity during extinction recall following MBSR and SME. This analysis yielded significant results for the MBSR intervention. Following mindfulness training, increases in left subiculum volume were associated with decreased coupling between the left hippocampus and two clusters in lateral occipital cortex, corresponding to the ventral visual stream (Figure 1b; MNI coordinates [48, −60, 00], *k* = 244, and MNI coordinates [44, −80, 10], *k* = 134; with voxel threshold *p* < .001 and a cluster threshold FWE corrected at *p* < .05) in the MBSR group.

### 3.4 Correlation between changes in hippocampal connectivity and changes in self‐reported anxiety

To investigate the potential clinical significance of this finding, we then tested the association between changes in connectivity and changes in anxiety scores using the previously reported difference in anxiety scores. For this exploratory analysis, only changes in self‐reported anxiety levels were used because it was the only outcome that differentiated the mindfulness intervention from the active control condition. We found a positive correlation between decreases in connectivity between the hippocampus and the cluster with peak MNI coordinates [48, −60, 00], and decreases in anxiety scores following mindfulness training, meaning that larger decreases in functional coupling were associated with larger decreases in anxiety levels (Figure 1b; *r* = .394, *p* = .021, *n* = 34).

## 4 DISCUSSION

Here, we postulated that the heightened attention to and awareness of sensory experience during mindfulness meditation creates an optimal therapeutic exposure condition and thereby improves extinction learning. In line with our previous reports of functional changes in hippocampal circuits during the contextual retrieval of extinction memory, here, we demonstrated an association between mindfulness training‐related increases in subiculum volume and decreased hippocampal connectivity to lateral occipital regions during contextual retrieval of extinguished fear. Further, we demonstrated an association between functional changes in the hippocampal connectivity and changes in anxiety following mindfulness training. While these results highlight the role of the subiculum in gating interactions with contextual stimuli during memory retrieval, they also provide a better understanding of the mechanisms through which mindfulness training fosters resilience.

One of the proposed mechanisms through which meditation practice promotes well‐being is extinction learning (Baer, 2003; Hölzel et al., 2011; Tang, Hölzel, & Posner, 2015). The turning of attention toward otherwise avoided sensory aspects of experience during meditation supports extinction learning through the formation of a new context‐dependent memory. The hippocampus plays a critical role in such safety learning, especially through the gating of context‐based newer safety memory during retrieval (Milad, Wright, et al., 2007). In line with the proposed mechanism of action, cross‐sectional studies report higher hippocampal gray matter concentrations in meditators compared to controls (Holzel et al., 2008; Luders et al., 2009). Similarly, longitudinal studies investigating mindfulness interventions have demonstrated increased hippocampal gray matter density (Holzel et al., 2011) as well as alterations in hippocampal functioning and increased connectivity between the hippocampus and other brain regions, both during and beyond meditative states (Brewer et al., 2011; Engström, Pihlsgård, Lundberg, & Söderfeldt, 2010; Garrison, Zeffiro, Scheinost, Constable, & Brewer, 2015; Kilpatrick et al., 2011; Taylor et al., 2013; Wang et al., 2011). Current results are in line with the conceptualization of extinction as an implicit form of emotion regulation (Ochsner, Silvers, & Buhle, 2012), and advance this conceptualization by reporting an association between mindfulness training‐dependent functional changes in hippocampal networks, and decrease in self‐reported anxiety levels.

The subiculum plays a critical role in memory consolidation and retrieval (Ledergerber & Moser, 2017), as well as in the modulation of the stress response through the hypothalamo–pituitary–adrenocortical axis (Herman & Mueller, 2006). The subiculum has also been associated with decreased glucocorticoid release (Herman et al., 1998; Herman & Mueller, 2006), and also in mediating the relation between stress and reward processing through the dopaminergic system (Valenti, Lodge, & Grace, 2011). Critically, an indirect pathway that involves the dorsal subiculum has been implicated in retrieval and in retrieval‐related functions such as rapid memory updating and modulation of retrieval‐driven fear responses (Roy et al., 2017).

Increased gray matter density within the subiculum has previously been reported in long‐term mindfulness practitioners compared to controls (Luders, Kurth, Toga, Narr, & Gaser, 2013). However, the functional significance of this transformation, especially in association with intervention‐related improvements, remained unknown. Here, we demonstrate an association between structural changes in the subiculum and retrieval of extinction memory following mindfulness training and propose a potential mechanism for mindfulness training‐induced reductions in anxiety.

In line with the reported modulation of neural activity in visual areas, exposure therapy has been previously associated with diminished visual responsiveness to phobogenic images (Hauner, Mineka, Voss, & Paller, 2012). Individual differences in the magnitude of visual cortex activations have been found to inversely predict long‐term therapeutic outcomes of exposure therapy (Hauner et al., 2012). Decreased functional connectivity between the hippocampus and regions of the ventral stream reported here may reflect alterations associated with context modulation of extinction memory (Milad, Orr, Pitman, & Rauch, 2005), potentially through memory updating or other retrieval‐related mechanisms (Roy et al., 2017). Such an interpretation is in line with the proposed overlap between mindfulness meditation and exposure contexts, as well as with the proposed mechanisms of change associated with mindfulness training (Hölzel et al., 2011; Tang et al., 2015).

The significant correlation between changes in anxiety and hippocampal functional coupling to ventral visual stream supports this interpretation and also may pinpoint a more generalized mindfulness training‐induced benefit in stress responses. In addition to its role in contextual retrieval, studies of memory‐guided orienting suggest that the hippocampus may have a proactive role in influencing our interaction with perceptual stimuli (Summerfield, Lepsien, Gitelman, Mesulam, & Nobre, 2006). An inability to process safety cues, including contextual information provided by the hippocampus, is a hallmark for trait anxiety (Haaker et al., 2015; Ziv, Tomer, Defrin, & Hendler, 2010) and has been implicated in the pathogenesis of anxiety disorders (Cui et al., 2016; Geng et al., 2018). The correlation between changes in anxiety levels and the functional changes in the processing of extinguished fear response to visual stimuli corroborates this interpretation and further postulates a potential subiculum‐mediated route for mindfulness training‐dependent changes in anxiety levels (Goldin & Gross, 2010; Hoge et al., 2013; Treanor, 2011).

These findings are also in line with the vulnerability of the subiculum to traumatic experience (Teicher, Anderson, & Polcari, 2012) and hippocampal–cortical reorganization following mindfulness training during extinction retrieval (Sevinc at al., 2019). Yet, it is important to note that the unequal allocation ratio may have limited our ability to detect effects in the control group due to the lower power. Future studies with an equal allocation ratio and using other metrics and/or criteria for computing an index of extinction retention (Marin et al., 2019) are needed to further explore the role of subiculum in improvements in extinction retrieval following mindfulness training. Nevertheless, the results of this longitudinal investigation provide the first evidence for an association between hippocampal subfields, particularly the subiculum, with training‐related improvements in context‐dependent retrieval of extinction and advocate hippocampal pathways as one mechanism through which enhanced extinction retrieval fosters resilience. Finally, given the relevance of extinction learning to exposure therapy, the results also suggest a novel way to enhance extinction learning in therapeutic settings.

## 5 CONCLUSION

Here, we examined the association between morphological changes in the subiculum and alterations in hippocampal connectivity during the retrieval of extinction memory following mindfulness training. The results revealed an association between alterations in subiculum volume and decreased hippocampal connectivity to lateral occipital regions during the retrieval of extinguished fear. An exploratory analysis further revealed a positive association between the magnitude of decreased hippocampal connectivity to visual association areas and decreases in self‐reported anxiety levels. These findings are in line with conceptualization of mindfulness meditation as a form of exposure and propose extinction learning as a potential mechanism for mindfulness training‐induced reductions in anxiety. Future studies are needed to investigate neural correlates of extinction learning following mindfulness‐based interventions both in healthy and patient populations and also using personally relevant fear‐inducing cues.

## CONFLICT OF INTEREST

The authors declare that the research was conducted in the absence of any commercial or financial relationships that could be construed as a potential conflict of interest.

## AUTHOR CONTRIBUTIONS

BKH, MRM, SWL, MV, and SPO conceived and planned the experiments. BKH, TG, and TC carried out data collection. GS and JG processed the experimental data, performed the analyses, and drafted the manuscript. SWL oversaw all data collection and analysis. BKH, TG, JAH, SPO, MRM, and SWL contributed to the interpretation of the results. All authors provided critical feedback and discussed the results and commented on the manuscript.

### Peer Review

The peer review history for this article is available at https://publons.com/publon/10.1002/brb3.1766.

## Data Availability

The data that support the findings of this study are available from the corresponding author upon reasonable request.
